# A Phase-Preserving Focusing Technique for TOPS Mode SAR Raw Data Based on Conventional Processing Methods

**DOI:** 10.3390/s19153321

**Published:** 2019-07-29

**Authors:** Adele Fusco, Antonio Pepe, Paolo Berardino, Claudio De Luca, Sabatino Buonanno, Riccardo Lanari

**Affiliations:** IREA—National Research Council of Italy (CNR) via Diocleziano 328, 80124 Napoli, Italy

**Keywords:** TOPS, SAR, Raw data, SAR focusing algorithms, Sentinel-1

## Abstract

We present a new solution for the phase-preserving focusing of synthetic aperture radar (SAR) raw data acquired through the Terrain Observation with Progressive Scan (TOPS) mode. The proposed algorithm consists of a first interpolation stage of the TOPS raw data, which takes into account the Doppler Centroid frequency variations due to the azimuth antenna steering function, and allows us to unfold the azimuth spectra of the TOPS raw data. Subsequently, the interpolated signals are processed by using conventional phase-preserving SAR focusing methods that exploit frequency domain and spectral analyses algorithms, which are extensively used to efficiently process Stripmap and ScanSAR data. Accordingly, the developed focusing approach is easy to implement. In particular, the presented focusing approach exploits one of the available frequency domain Stripmap processing techniques. The only modification is represented by the inclusion, within the 2D frequency domain focusing step, of a spurious azimuth chirp signal with a properly selected azimuthal rate. This allows us to efficiently carry out the TOPS azimuth focusing through the SPECAN method. Furthermore, an important aspect of this algorithm is the possibility to easily achieve a constant and tunable output azimuth pixel size without any additional computing time; this is a remarkable feature with respect to the full-aperture TOPS-mode algorithms available in the existing literature. Moreover, although tailored on Sentinel-1 (S1) raw data, the proposed algorithm can be easily extended to process data collected through the TOPS mode by different radar sensors. The presented experimental results have been obtained by processing real Sentinel-1 raw data and confirm the effectiveness of the proposed algorithm.

## 1. Introduction

Synthetic Aperture Radar (SAR) is an active microwave sensor which presently plays a fundamental role in the Earth observation scenario [1]. Spaceborne SAR systems typically have the capability to operate with the conventional Stripmap and ScanSAR imaging modes [2,3,4]. However, the rising demands in having wider swath coverage and/or finer azimuth resolution have led to the development of new advanced imaging modes with improved performance. In particular, the capability to steer the radar antenna beam along the azimuth direction has led to the design of the Spotlight [5,6] and the Terrain Observation by Progressive Scans (TOPS) [7] modes, which are used to achieve enhanced azimuth resolution or wide-swath coverage, respectively. Notably, the TOPS mode is extensively exploited by the Sentinel-1 constellation [8,9], and it is going to be adopted as principal acquisition mode for wide-swath imaging of the TerraSAR-X2 [10] and the Chinese spaceborne SAR missions [11,12]. As with the conventional ScanSAR mode [13], the TOPS imaging mode achieves wide-swath coverage by switching the antenna beam along range direction from swath to swath (often referred to as sub-swaths), but it is able to achieve better azimuth resolution and to overcome the major ScanSAR drawbacks, i.e., the azimuth-varying signal-to-noise ratio and ambiguity-to-signal ratio as well as the so-called “scalloping” effect, introduced by the antenna steering [7,13]. These effects are undesired and require an appropriate post-processing filtering stage to compensate them in the focused ScanSAR images [14,15,16].

For what concerns the TOPS mode (at variance with ScanSAR) the antenna beam rotates along azimuth throughout the acquisition from backward to forward, i.e., with the opposite direction to the Spotlight case, and this leads all targets to be illuminated during the acquisition data duration (burst) within a large portion of the azimuth antenna pattern, thus mitigating the above-mentioned SAR image quality degradation effects. A consequence of the TOPS operating mode is that targets located at different azimuth positions are imaged with different squint angles; this implies that the resulting azimuth signals have a larger bandwidth, much greater than the pulse repetition frequency (PRF) used in the Stripmap case. However, because the PRF is maintained the same as the Stripmap mode, the Doppler spectra result aliased. Therefore, the conventional SAR focusing methods implemented in the frequency domain, such as the range-Doppler algorithm (RDA) [17,18], the chirp scaling algorithm (CSA) [19,20], the extended CSA algorithm [21], the Omega-K algorithm [22,23], the 2-D Fast Fourier Transform algorithm [24,25,26,27] and the chirp-Z transform algorithm [28,29], are not directly applicable for efficiently focusing TOPS raw data.

In recent years, several imaging methods have been developed to overcome these problems and to process TOPS raw datasets [30,31,32,33,34,35,36,37,38,39]. A first group of these algorithms [30,31] relies on the use of sub-apertures. In this case, each burst raw dataset is split into several azimuth blocks (partially overlapped) wherein the PRF is higher than the instantaneous azimuth bandwidth; this allows one to correct the range cell migration (RCM) in each sub-aperture by using one of the above-mentioned Stripmap focusing algorithms based on spectral analyses. However, the major drawback in using sub-apertures is that usually a large number of overlapped small azimuth blocks must be introduced. Accordingly, by also considering the subsequent recombination steps of the sub-apertures and the matched filter used to focus the full-aperture data, the methods based on sub-apertures turn out to be precise but not particularly computationally efficient.

Alternatively, to the sub-aperture methods, full-aperture imaging algorithms have also been proposed [7,30,31,32,33,34,35,36,37,38,39]. These techniques are effective but, on the other hand, they require significant modifications with respect to conventional spectral-based Stripmap focusing techniques, and may request the implementation of rather complex additional processing stages. Among these, we remark that an efficient solution is the one presented in [39] which, however, requests a non-trivial modification of the original chirp-Z transform approach to implement the referred moving band chirp-Z transform (MBCZT).

In this paper, we present a new solution for a precise and efficient focusing of TOPS raw data. The proposed algorithm satisfies the phase quality requirements for interferometric applications [40,41], i.e., it belongs to the class of phase-preserving focusing algorithms [42,43]. In particular, the presented approach is based on a first interpolation stage of the range-compressed SAR data, which is accomplished by taking into account the Doppler frequency variation due to the antenna rotation along azimuth direction, and permits to effectively unfold the azimuthal spectra. Subsequently, the range-compressed data are fully focused by using conventional frequency domain and spectral analyses focusing approaches which have been extensively exploited to focus Stripmap and ScanSAR raw data [24,25,26,27,44]. Accordingly, the presented TOPS raw data focusing approach is easy to implement. Furthermore, a significant feature of the developed focusing method is the possibility to have a tunable selection of the azimuth pixel dimension of the focused SAR images. This is achieved by artificially introducing, before azimuth compression, a spurious azimuth chirp signal in the 2-D frequency domain, which is subsequently compensated for through the SPECAN approach [44].

The paper is organized as follows. Section 2 addresses the TOPS acquisition geometry and the TOPS signal characteristics. Section 3 describes the proposed TOPS focusing algorithm. Section 4 shows some examples achieved by applying the developed focusing algorithm to Sentinel-1 raw data acquired through the TOPS mode. Discussion and conclusions are finally drawn in Section 5.

## 2. TOPS Acquisition Mode and System Transfer Function Analysis

**Figure 1:**
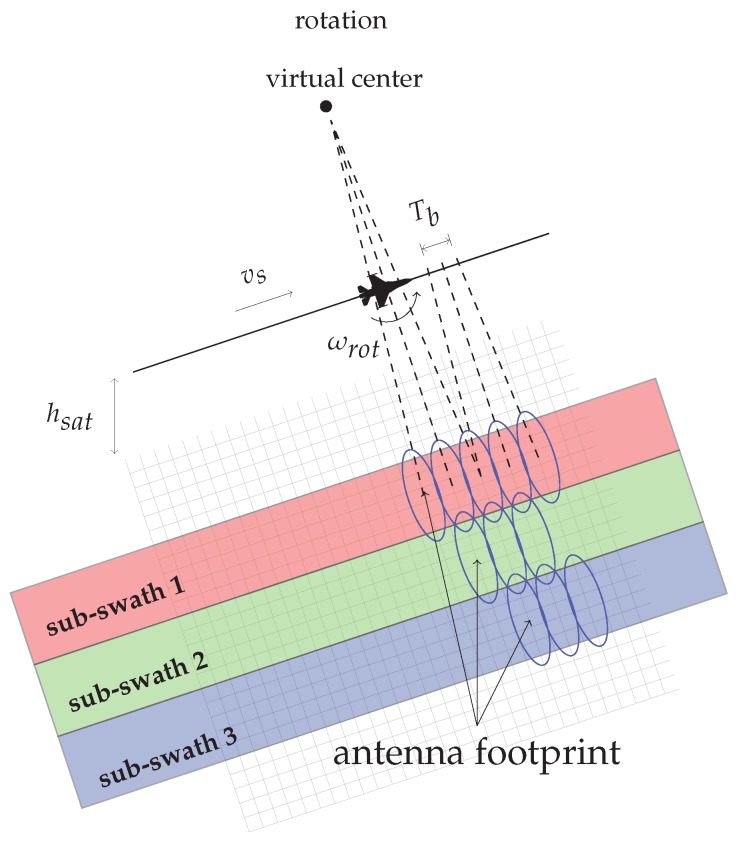
TOPS acquisition mode: the antenna beam has a virtual rotation center located above the platform acquisition track and an angular velocity $\omega_{rot}$. TOPS raw data are acquired in bursts of duration $T_{b}$, cyclically switching the antenna beam from swath to swath, referred to as sub-swaths, for wide-area coverage. Note also that $v_{s}$ and $h_{sat}$ represent the platform velocity and height, respectively.

The TOPS SAR acquisition mode operates by electronically steering the azimuth beam position from backward to forward, with an antenna angular velocity, namely $\omega_{rot}$, with respect to a virtual rotation center (see Figure 1). Moreover, similarly to the ScanSAR mode [2], the TOPS raw data are acquired in bursts, say of duration $T_{b}$, by cyclically changing the antenna beam between adjacent sub-swaths (see Figure 1).

**Figure 2:**
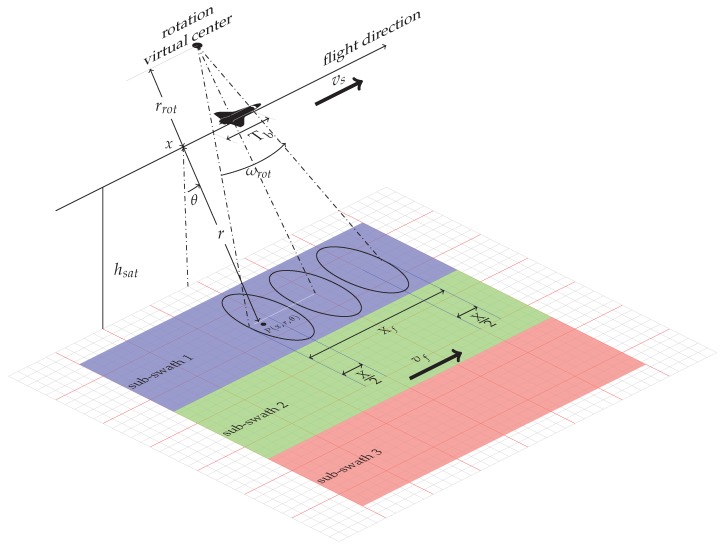
TOPS acquisition geometry for a single burst: P≡P(x,r,θ) represents the location of a generic target, $v_{s}$ is the sensor velocity, $v_{f}$ the antenna footprint velocity, $\omega_{rot}$ the angular rotation velocity, $r_{rot}$ the distance of the SAR sensor flight track from the virtual rotation center, $T_{b}$ the acquisition burst time, $X_{f}$ the illuminated area extension on the ground, *X* the azimuth antenna footprint.

Figure 2 describes the TOPS SAR geometry for a single burst, where the position of the generic target P≡P(x,r.θ) is defined with respect to a cylindrical coordinates system, whose principal axis *x* corresponds to the platform flight direction, whereas *r* represents the range coordinate of the target and θ is the side looking angle.

As evident from Figure 2, due to the antenna azimuth rotation, there is a difference between the platform velocity $v_{s}$ and the antenna footprint velocity $v_{f}$. In particular, we have:

(1) $$v_{f}≃\omega_{rot}\left(r+r_{rot}\right)=\omega_{rot}r+v_{s}.$$

Through simple geometric considerations (see Figure 2), we can derive the illuminated area extension on the ground $X_{f}$ (see Equation (2)) as follows:

(2) $$X_{f}≃T_{b}v_{f}+X$$

where X=λr/L represents the azimuth antenna footprint extension on the ground, being λ the operational wavelength and *L* the azimuth antenna length. Please note that the ground illuminated area extension X in Equation (2) is typically considered at mid-range. Note also that due to the antenna azimuth rotation, the Doppler Centroid variation with respect to time DC(t) has the following expression:

(3) $$DC\left(t\right)=-\frac{2v_{s}}{\lambda }\sin\left(\psi_{DC}\left(t\right)\right)$$

where $\psi_{DC}\left(t\right)=\omega_{rot}t$, t being the time variable. We further remark that the Doppler Centroid value could also be influenced by a possible squint angle; however, this is neglected in the following analysis.

Let us now concentrate on the derivation of analytical expression of the System Transfer Function (STF) for the TOPS mode, where for the sake of simplicity, inessential amplitude factors are neglected.

We first assume that the SAR system transmits, at time $t_{n}-\tau /2$, a linearly frequency modulated (FM) chirp, which can be represented by using the complex formalism as follows [3]:

(4) $$s\left(t-t_{n}\right)=\exp\left[j2\pi f(t-t_{n})\right]\exp\left[-j\frac{\alpha }{2}{\left(t-t_{n}\right)}^{2}\right]rect\left[\frac{t-t_{n}}{\tau }\right]$$

where *f* is the carrier frequency, τ is the pulse duration, and α=2πΔf/τ is the chirp rate, Δf being the transmitted chirp bandwidth. Accordingly, the raw data signal received onboard, related to the point target of radar coordinates P≡P(x,r), can be expressed as follows:

(5) $$\begin{array}{c} \begin{array}{cc} s_{rec}\left(t-t_{n};x,r\right)= & \exp\left[j2\pi f\left(t-t_{n}-\frac{2R}{c}\right)\right]\exp\left[-j\frac{\alpha }{2}{\left(t-t_{n}-\frac{2R}{c}\right)}^{2}\right]\\ & rect\left[\frac{t-t_{n}-2R/c}{\tau }\right]W_{a}^{2}\left[\frac{t-t_{n}-\frac{x}{v_{f}}}{\frac{X}{v_{f}}}\right]rect\left[\frac{t-t_{n}}{T_{b}}\right]rect\left[\frac{x}{X_{f}}\right] \end{array} \end{array}$$

where, as said, we assume that there is no squint angle and $R=\sqrt{r^{2}+{\left(v_{s}\left(t-t_{n}\right)-x\right)}^{2}}=r+\Delta R$, is the sensor-to-target distance. The received signal includes the two-way azimuth antenna gain $W_{a}^{2}\left[\cdot \right]$ as well as the effect of the bursting operation due to the TOPS acquisition mode and of the illuminated area extension on the ground.

By neglecting the fast-varying term $\exp\left[j2\pi f(t-t_{n})\right]$, which is compensated by the heterodyne receiver, and introducing the range $r^{\prime }=c\left(t-t_{n}\right)/2$ and azimuth $x^{\prime }=v_{s}\left(t-t_{n}\right)$ spatial variables, since the *light velocity* is c=λf, Equation (5) becomes:

(6) $$\begin{array}{c} \begin{array}{cc} s_{rec}\left(x^{\prime },r^{\prime };x,r\right)= & \exp\left(-j4\pi r/\lambda \right)\exp\left(-j4\pi \frac{\Delta R}{\lambda }\right)\exp\left[-j\frac{2\alpha }{c^{2}}{\left(r^{\prime }-\left(r+\Delta R\right)\right)}^{2}\right]\\ & rect\left[\frac{r^{\prime }-\left(r+\Delta R\right)}{c\tau /2}\right]rect\left[\frac{\frac{x^{\prime }}{v_{s}}-\frac{x}{v_{f}}}{\frac{X}{v_{f}}}\right]rect\left[\frac{\frac{x^{\prime }}{v_{s}}}{\frac{X_{b}}{v_{s}}}\right]rect\left[\frac{x}{X_{f}}\right] \end{array} \end{array}$$

where $X_{b}=v_{s}\cdot T_{b}$ is the spatial extension of the flight trajectory during the acquisition burst time $T_{b}$. Please note that in Equation (6) the constant phase term $\exp\left(-j4\pi r/\lambda \right)$ represents the phase contribution that is explored by the SAR interferometry techniques [3], and the two-way azimuth antenna gain has been approximated with a rect function, i.e., $W_{a}^{2}\left[\cdot \right]=rect\left[\cdot \right]$.

Moreover, it can be easily shown that:

(7) $$\frac{x^{\prime }}{v_{s}}-\frac{x}{v_{f}}=\frac{1}{v_{s}}\left(x^{\prime }-\frac{x}{A}\right)$$

where based on Equation (1):

(8) $$A=\frac{v_{f}}{v_{s}}=\frac{\omega_{rot}r+v_{s}}{v_{s}}=\frac{r+r_{rot}}{r_{rot}}>1$$

Accordingly, considering Equations (6)–(8), the TOPS SAR burst impulse response has the following expression:

(9) $$g^{\prime }\left(x^{\prime }-x,r^{\prime }-r;x,r\right)=\exp\left(-j4\pi r/\lambda \right)rect\left[\frac{x}{X_{f}}\right]g\left(x^{\prime }-x,r^{\prime }-r,x,r\right)$$

where $g(x^{\prime }-x,r^{\prime }-r,x,r)$ is:

(10) $$\begin{array}{c} \begin{array}{cc} g(x^{\prime }-x,r^{\prime }-r,x,r)= & \exp\left[-j\frac{2\alpha }{c^{2}}{\left(r^{\prime }-\left(r+\Delta R\right)\right)}^{2}\right]rect\left[\frac{r^{\prime }-\left(r+\Delta R\right)}{c\tau /2}\right]\\ & \exp\left[-j4\pi \frac{\Delta R}{\lambda }\right]rect\left[\frac{x^{\prime }-\frac{x}{A}}{\frac{X}{A}}\right]rect\left[\frac{x^{\prime }}{X_{b}}\right] \end{array} \end{array}$$

At this stage, the TOPS transfer function can be obtained by Fourier transforming Equation (10):

(11) $$G\left(\xi ,\eta ;x,r\right)=\int \int dx^{\prime }dr^{\prime }g\left(x^{\prime }-x,r^{\prime }-r;x,r\right)e^{-j2\pi \xi (x^{\prime }-x)}e^{-j2\pi \eta (r^{\prime }-r)}$$

where ξ and η denote the azimuth and range spatial frequencies, respectively. Due to the rather large time-bandwidth product of the received signal, the integration in Equation (11) can be carried out via the *stationary phase* method [3,24] thus obtaining, after trivial calculations that:

(12) $$G\left(\xi ,\eta ;x,r\right)=G_{RG}\left(\eta \right)G_{TOPS}\left(\xi ,\eta ;x,r\right)$$

where the term:

(13) $$G_{RG}\left(\eta \right)\approx rect\left[\frac{\eta }{2\Delta f/c}\right]\exp\left[-j2\pi \frac{c^{2}\tau }{8\Delta f}\eta^{2}\right]$$

can be straightforwardly compensated during range compression focusing step [2,3,4] and:

(14) $$G_{TOPS}\left(\xi ,\eta ;x,r\right)\approx rect\left[\frac{\xi -\frac{2x}{\lambda r}\left(1-\frac{1}{A}\right)}{\frac{2}{L_{e}}}\right]rect\left[\frac{\xi -\frac{2}{\lambda r}x}{\frac{2X_{b}}{\lambda r}}\right]\exp\left[-jrk(\xi ,\eta )\right]$$

wherein:

(15) $$L_{e}=AL>L$$

and

(16) $$k\left(\xi ,\eta \right)=2\pi \left[\left(\frac{2}{\lambda }+\eta \right)-\sqrt{\left(\frac{2}{\lambda }+\eta \right)-\xi^{2}}\right]\approx \frac{\pi \xi^{2}\lambda }{2}-\frac{\pi \xi^{2}\eta \lambda^{2}}{4}$$

We also observe that in Equation (14) the windowing function $rect\left[\left(\xi -\frac{2}{\lambda r}x\right)/\frac{2X_{b}}{\lambda r}\right]$ is inessential for the following analysis, because $2X_{b}/\lambda r>2/L_{e}$ and therefore, it has been neglected hereinafter.

We further remark that the term rk(ξ,η) in Equation (14) can be written as follows [3,25]:

(17) $$rk\left(\xi ,\eta \right)=r_{0}k\left(\xi ,\eta \right)+\left(r-r_{0}\right)k\left(\xi ,\eta \right)$$

Accordingly, Equation (14) can be factorized into a range invariant and range variant phase signal component, as follows:

(18) $$G_{TOPS}\left(\xi ,\eta ;x,r\right)=G_{0}\left(\xi ,\eta ;x,r_{0}\right)\cdot G_{\Delta }\left(\xi ,\eta ;r-r_{0}\right)$$

where considering Equation (16), we have:

(19) $$\begin{array}{ccc} G_{0}\left(\xi ,\eta ;x,r,r_{0}\right) & = & rect\left[\frac{\xi -\frac{2x}{\lambda r}\left(1-\frac{1}{A}\right)}{\frac{2}{L_{e}}}\right]\exp\left[-jr_{0}k\left(\xi ,\eta \right)\right]≃\\ & ≃ & rect\left[\frac{\xi -\frac{2x}{\lambda r}\left(1-\frac{1}{A}\right)}{\frac{2}{L_{e}}}\right]\exp\left[-jr_{0}\left(\frac{\pi \xi^{2}\lambda }{2}\right)\right]\exp\left[jr_{0}\left(\frac{\pi \xi^{2}\eta \lambda^{2}}{4}\right)\right] \end{array}$$

(20) $$\begin{array}{ccc} G_{\Delta }\left(\xi ,\eta ;r-r_{0}\right) & = & \exp\left[-j\left(r-r_{0}\right)k\left(\xi ,\eta \right)\right]≃\\ & ≃ & \exp\left[j\left(r-r_{0}\right)\cdot \left(\frac{\pi \xi^{2}\lambda }{2}-\frac{\pi \xi^{2}\eta \lambda^{2}}{4}\right)\right]. \end{array}$$

Please note that the approximations presented in Equations (19) and (20) are based on the Taylor expansion in Equation (16) and are rather typical in the SAR Literature. However, they are not essential for the following analysis.

Based on Equations (14), (15) and (19), some considerations are now in order. First, we note that the TOPS azimuth (spatial) spectrum is centered around the Doppler Centroid spatial frequency:

(21) $$\xi_{Dopp}^{\prime }\left(x,r\right)=\frac{2x(1-1/A)}{\lambda r}$$

which depends on the angular rotation velocity accounted by *A* and on the azimuth *x* and range *r* position of the target. We further note that $L_{e}$, defined in Equation (15), represents a sort of equivalent antenna length for the TOPS mode, which is larger then that of the Stripmap one, thus implying a sort of “shrinking” of the antenna footprint [7].

Accordingly, it is evident that the point-target azimuth TOPS signal spatial bandwidth, which is equal to:

(22) $$B_{f}=2/L_{e}$$

is reduced with respect to the point-target azimuth Stripmap signal spatial bandwidth, which is equal to 2/L [1,2,3,4], thus leading to an azimuth resolution degradation:

(23) $$\Delta x_{TOPS}^{\prime }=\frac{L_{e}}{2}=\frac{AL}{2}>\frac{L}{2}.$$

**Figure 3:**
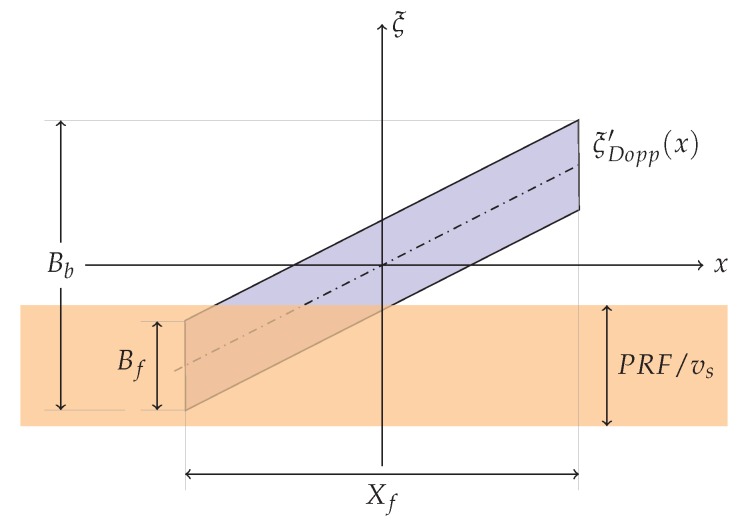
Raw data space/(spatial) frequency representation for the TOPS mode: $B_{f}$ represents the bandwidth for a single point target, $B_{b}$ is the overall bandwidth, $PRF/v_{s}$ is the spatial azimuth pulse repetition frequency, $\zeta_{Dopp}^{\prime }\left(x\right)$ is the Doppler Centroid (spatial) frequency considered here as a function of the azimuth coordinate x.

Moreover, based on from Equations (14) and (21), the total azimuth bandwidth of a single burst $B_{b}$ can be derived as follows:

(24) $$\begin{array}{ccc} B_{b} & ≃ & \xi_{Dopp}^{\prime }\left(\frac{X_{f}}{2}\right)-\xi_{Dopp}^{\prime }\left(-\frac{X_{f}}{2}\right)+B_{f}\\ & = & \frac{2X_{f}}{\lambda r}\left(1-\frac{1}{A}\right)+B_{f} \end{array}$$

as pictorially shown in Figure 3.

As previously stated, we remark that the TOPS raw data are sampled along the azimuth direction by considering a PRF that is consistent with the Stripmap mode bandwidth [2,45], whose spatial expression is $PRF/v_{s}$.

Because:

(25) $$\frac{PRF}{v_{s}}\geq \frac{2}{L}>\frac{2}{L_{e}}=B_{f}$$

and

(26) $$\frac{PRF}{v_{s}}<B_{b}$$

it turns out that for the TOPS mode the used PRF is greater than the azimuth bandwidth of a single point target ($B_{f}$) but it is significantly smaller of the overall bandwidth ($B_{b}$), as pictorially shown in Figure 3.

Accordingly, the TOPS raw data are not correctly sampled at PRF, thus introducing an azimuth spectrum folding. This issue prevents the straightforward application of one of the several computational efficient phase-preserving Stripmap focusing techniques implemented in the frequency domain [17,18,19,20,21,22,23,24,25,26,27,28,29]. We propose in the following a simple but effective focusing approach, which relies on the above-mentioned frequency-based Stripmap focusing approaches and on the spectral analysis algorithm referred to as SPECAN method [44].

## 3. Focusing Algorithm

**Figure 4:**
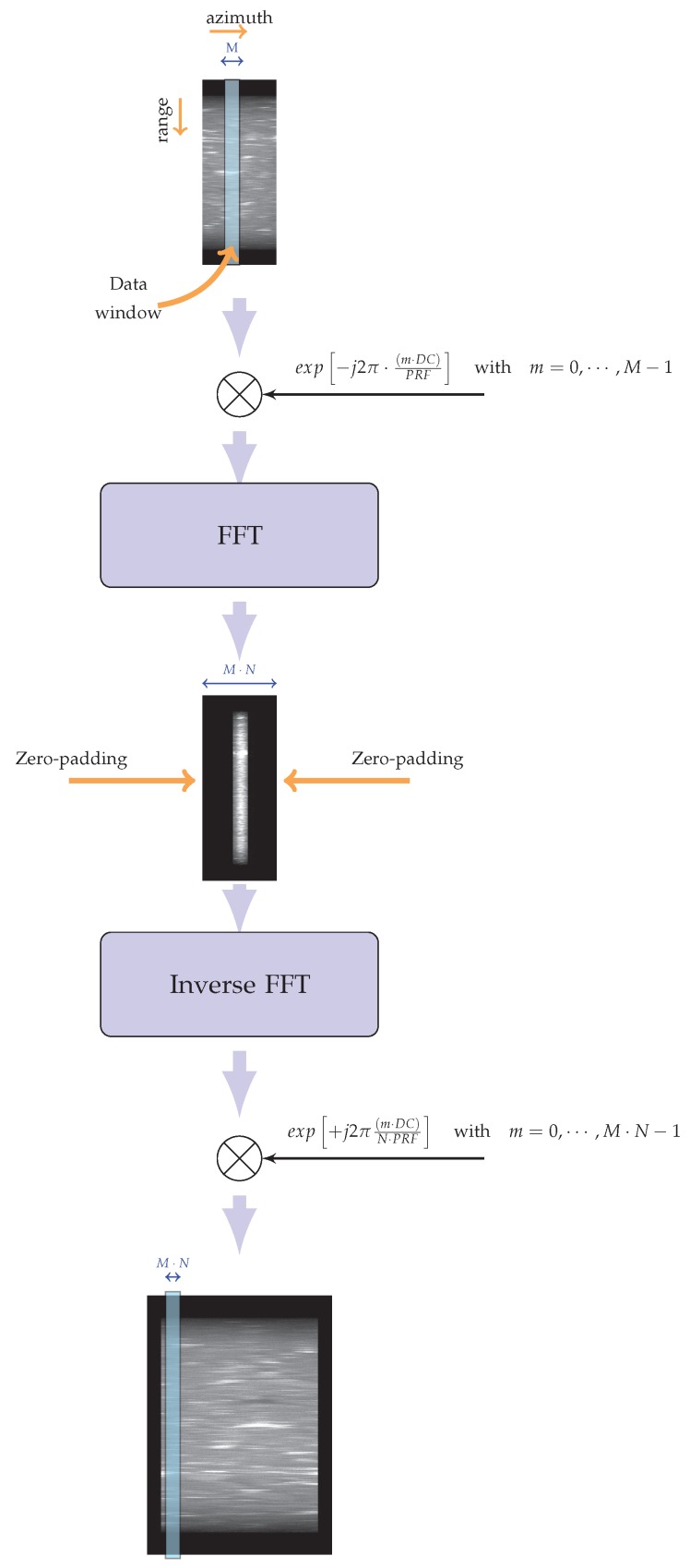
Flow chart of the implemented azimuth interpolation. Please note that PRF is the pulse repetition frequency, DC is the Doppler Centroid, *M* is the length of the sliding window, and *N* is the oversampling factor.

Let us start this analysis by considering a straightforward implementation, although computationally inefficient, of a TOPS-mode focusing approach. To do this, let us first consider the range-compressed TOPS raw data, for which the $G_{RG}\left(\eta \right)$ factor (see Equation (13)) is compensated for in the range frequency domain [2,3,4,5]. Subsequently, an azimuthal interpolation is carried out, considering an oversampling factor $N=\lceil B_{b}/PRF\rceil$, ⌈·⌉ being the ceil operator. Please note that for the TOPS Interferometric Wide-Swath (IWS) mode of the Sentinel-1 A/B sensors *N* is equal to 5. We further remark that in our implementation the azimuth interpolation is carried out locally by using a sliding window, say of length *M* (typically equal to 16 samples), to select portions of the raw data samples to be interpolated; in addition, we use Fast Fourier Transform (FFT) codes [46] to achieve high computational efficiency. The implemented procedure is shown in Figure 4.

**Figure 5:**
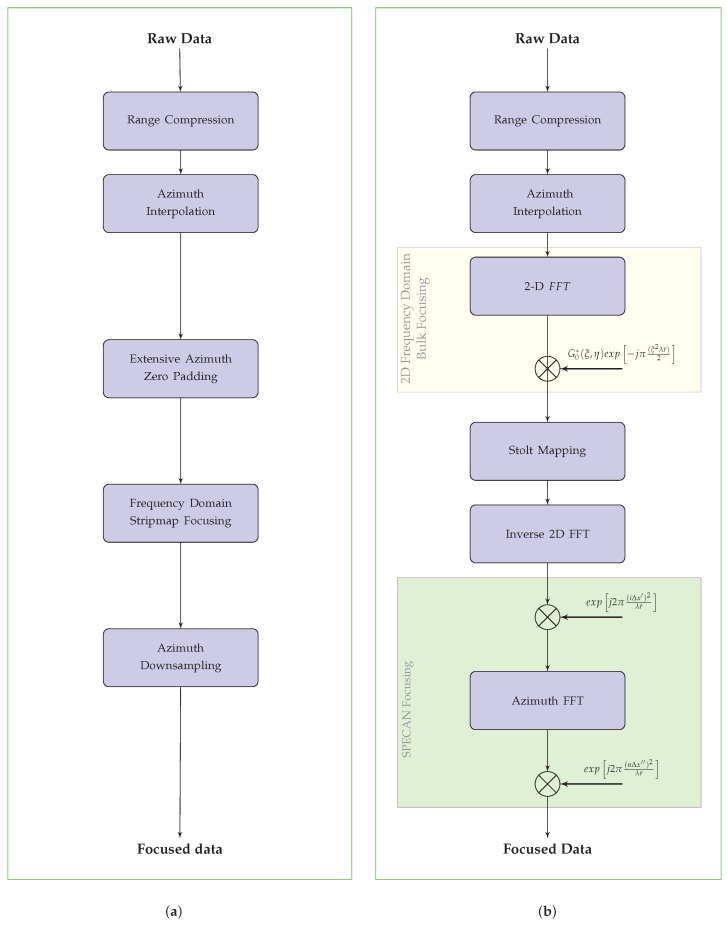
(**a**) Flow chart of a straightforward Stripmap-based TOPS raw data focusing algorithm; (**b**) Flow chart of the proposed TOPS raw data focusing algorithm.

First of all, for each data block selected by the applied data window, say of length *M*, the azimuthal frequencies are shifted to zero. This is achieved by multiplying the data with a complex exponential function accounting for the Doppler Centroid value (see Equation (3)) at the center of the selected block. Subsequently, the data can be straightforwardly interpolated through the cascade of an FFT step, a zero-padding block and an inverse FFT [47]. Finally, to restore the phase information of the interpolated signal, we multiply the data with the same complex exponential function used before but with an opposite sign and an increased sampling, accounting for the oversampling factor. Please note that by using a constant Doppler Centroid, it will result in a slight azimuth spectrum aliasing, which is removed by simply using an azimuth bandwidth filter. We further remark that results similar to what we achieve through our interpolation scheme can be obtained by applying the approach based on the spectrum replication and filtering presented in [7]. However, the solution presented in our work is efficient and very simple to implement (see Figure 5). Because the oversampled data have now unfolded azimuth spectra, a conventional Stripmap focusing algorithm [17,18,19,20,21,22,23,24,25,26,27,28,29] might straightforwardly be applied. Nonetheless, this strategy is not effective in this case, because to correctly represent each focused burst image, a very large zero padding would be required. Indeed, the number of focused azimuth pixels for each burst image, namely $N_{focused}$, would be much greater than the number of the interpolated raw data azimuth pixels, say $N_{interp}$, given by:

(27) $$\begin{array}{ccc} N_{interp} & = & T_{b}\cdot PRF_{TOPS}=\frac{X_{B}}{v_{s}}N\cdot PRF=\frac{X_{B}}{\Delta x^{\prime }} \end{array}$$

(28) $$\begin{array}{ccc} N_{focused} & = & T_{f}\cdot PRF_{TOPS}=\frac{X_{f}}{v_{s}}N\cdot PRF=\frac{X_{f}}{\Delta x^{\prime }} \end{array}$$

thus leading to have

(29) $$N_{focused}=\frac{X_{f}}{X_{B}}\cdot N_{interp}>>N_{interp}\;\;\mathrm{being}\;X_{f}>>X_{B}$$

where $\Delta x^{\prime }=v_{s}/PRF_{TOPS}=v_{s}/\left(N\cdot PRF\right)$ is the output azimuth pixel spacing. To clarify this issue we present in Table 1 typical $N_{\mathrm{interp}}$ and $N_{\mathrm{focused}}$ values for the sub-swath-1, sub-swath-2 and sub-Swath-3 of the TOPS Interferometric Wide-Swath (IWS) mode of the Sentinel-1 A/B sensors, referred as IW1,IW2, and IW3, respectively.

Accordingly, we can clearly argue that the straightforward use of Stripmap focusing algorithms (see Figure 5a) is quite inefficient for processing raw data acquired through the TOPS mode. In the following, we propose an alternative, efficient approach for an effective exploitation of conventional spectral analysis-based focusing methods.

To do this, we consider the 2-D Fourier transform algorithm [24,25,26,27] where, following the previously mentioned azimuth interpolation step, a bulk focusing operation is first performed in 2-D frequency domain. By referring to Equation (19), this operation consists of the compensation of the complex conjugate of the signal $G_{0}\left(\xi ,\eta ;x,r_{0}\right)$ shown in Equation (20), within the overall azimuth bandwidth $B_{b}$ (see Equation (24)).

Moreover, at the same time, we artificially introduce a spurious azimuth chirp signal in the 2-D frequency domain. This is done by multiplying the 2-D spectrum of the compensated bulk-focused TOPS raw signal by the following phase term:

(30) $$Z\left(\xi \right)=\exp\left[-j\tilde{r}\left(\frac{\pi \xi^{2}\lambda }{2}\right)\right]$$

where $\tilde{r}$ is a properly chosen range value, whose selection is clarified in the following. Please note that due to the spurious azimuth chirp signal, the raw data will remain unfocused along the azimuth direction and, therefore the extended azimuth zero padding needed for a conventional Stripmap focusing is not needed in our case. At this stage, following the burst focusing step implemented in 2-D frequency domain, the Stolt mapping operation [48] (which can be implemented in several ways, see [18,19,23,25,28]) is applied, and a 2-D inverse FFT step, is then performed, leading to the signal $\tilde{s}\left(x^{\prime },r^{\prime };x,r\right)$. The spurious *defocusing term* (see Equation (30)) is finally compensated by carrying out the convolution, with respect to the azimuth direction, between the signal $\tilde{s}\left(x^{\prime }-x;x,r\right)$ and the relevant chirp signal:

(31) $$\zeta \left(x^{\prime };\tilde{r}\right)=\exp\left[j2\pi \frac{x^{\prime 2}}{\lambda \tilde{r}}\right]$$

representing the azimuth inverse Fourier transform of the factor in Equation (30). This convolution operation, expressed as follows:

(32) $$\tilde{s}\left(x^{\prime }-x,r^{\prime };x,r\right){⊛}_{az}\zeta \left(x^{\prime };\tilde{r}\right)$$

is efficiently performed through the SPECAN method [44]. Let us investigate the implementation of this azimuth convolution operation as detailed in [49]. Please note that in this case we refer to a discrete time implementation because it is particularly relevant for our analysis. Hence, we have that for an isolated target of radar coordinates (x,r), the output signal is:

(33) $$\begin{array}{ccc} \bar{s}\left(n\Delta x^{\prime \prime };x,r\right) & = & \sum_{i=0}^{N_{interp-1}}\tilde{s}\left(i\Delta x^{\prime };x,r\right)\zeta \left(n\Delta x^{\prime \prime }-i\Delta x^{\prime }\right)=\\ & = & \exp\left[j2\pi \frac{{\left(n\Delta x^{\prime \prime }\right)}^{2}}{\lambda \tilde{r}}\right]\sum_{i=0}^{N_{interp}-1}\tilde{s}\left(i\Delta x^{\prime };x,r\right)\exp\left[j2\pi \frac{{\left(i\Delta x^{\prime }\right)}^{2}}{\lambda \tilde{r}}\right]\cdot \\ & & \cdot \exp\left[-j2\pi \frac{2\Delta x^{\prime }\Delta x^{\prime \prime }}{\lambda \tilde{r}}\cdot i\cdot n\right]\;\;\;\;\;with\;\;n=0,\cdots ,N_{interp}-1 \end{array}$$

where $\Delta x^{\prime }$ and $\Delta x^{\prime \prime }$ are the raw data (interpolated) and the focused image azimuth pixel spacings, respectively. Accordingly, the convolution operation is accomplished by the cascade of three stages. First, the signal $\tilde{s}(\cdot$) is multiplied by the de-ramping term:

(34) $$\exp\left[j2\pi \frac{{\left(i\Delta x^{\prime }\right)}^{2}}{\lambda \tilde{r}}\right]$$

Second, a DFT operation is performed trough the computationally efficient FFT algorithm [46]; to this aim the range value $\tilde{r}$ has to be chosen as follows:

(35) $$2\frac{\Delta x^{\prime }\Delta x^{\prime \prime }}{\lambda \tilde{r}}=\frac{1}{N_{interp}}.$$

Finally, the achieved result is multiplied by the residual phase $\exp\left[j2\pi {\left(n\Delta x^{\prime \prime }\right)}^{2}/\lambda \tilde{r}\right]$ and the focused burst is obtained.

The above analysis shows that through an appropriate selection of the $\tilde{r}$ factor of an artificially introduced spurious azimuth chirp, we may exploit a conventional Stripmap algorithm followed by a SPECAN operation, leading out to focused TOPS image bursts without any extensive zero padding of the TOPS raw data. We further remark that the achieved algorithm simplicity justifies some efficiency loss due to the implemented raw data oversampling step (see Figure 4). As further remark, we underline that the burst-focused SAR image is usually under-sampled by a factor, say $N_{under}$, to reduce the amount of data to be handled.

**Figure 6:**
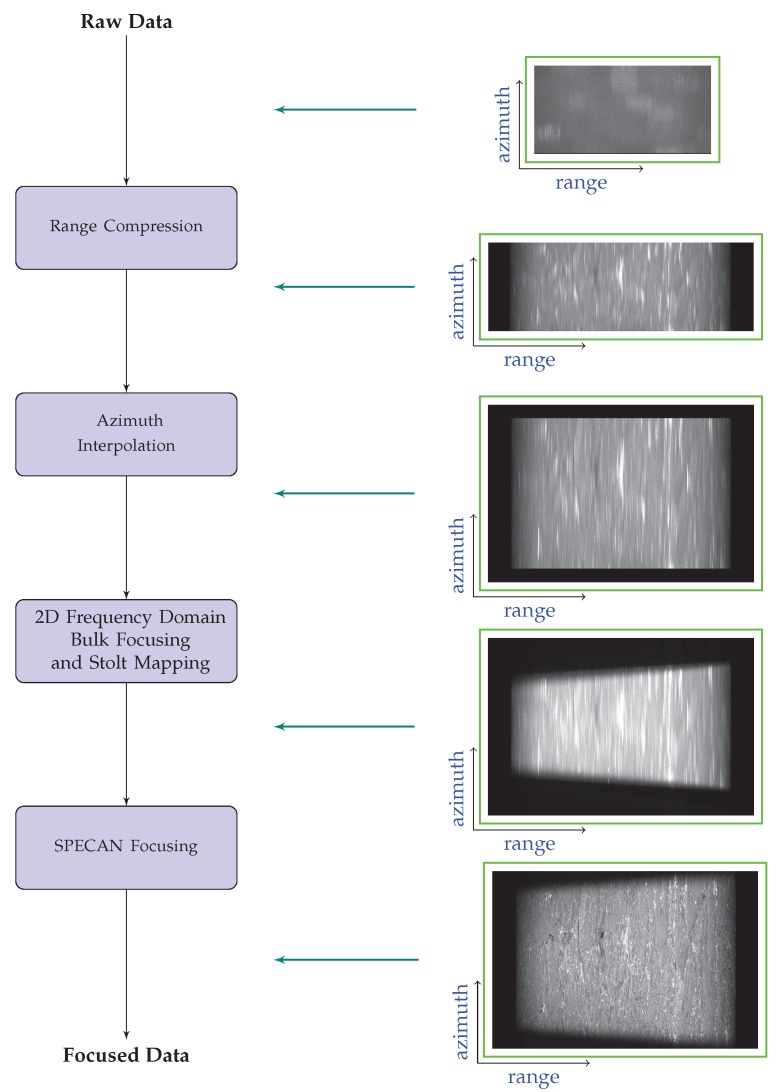
Flow chart of the proposed TOPS raw data focusing algorithm (see Figure 5b), showing the effect of each processing step on the data, starting from raw up to focused data. Please note that the extra zeros shown in the central panel of the Figure have been added only to have an azimuth dimension as power of 2 to efficiently perform the FFT operations.

Figure 5b synthesizes the block diagram of the algorithm presented in this section. Additionally, Figure 6 shows the effect of each processing step of the presented algorithm on the data, starting from raw up to focused data. We further remark that the final step of the presented has some similarities with the MBCZT operation proposed in [39]. Moreover, we also remark that the proposed approach has the interesting degree of freedom to allow the selection of the output pixel spacing $\Delta x^{\prime \prime }$, see Equation (35). This is an interesting feature which characterizes the presented approach with respect to most of the full-aperture TOPS-mode imaging algorithms available in the existing literature [30,31,32,33,34,35,36,37,38,39]. We finally underline that the application of a Stripmap processing approach different from that considered here (see [24,25,26,27]) would have no impact on the validity of the proposed TOPS raw data focusing algorithm.

## 4. Experimental Results from Sentinel-1 IWS Data

This section illustrates the achieved results obtained by applying the presented focusing algorithm, shown in Figure 5b and Figure 6, to Sentinel-1 raw data acquired through the TOPS IWS mode, with the main parameters summarized in Table 2.

**Figure 7:**
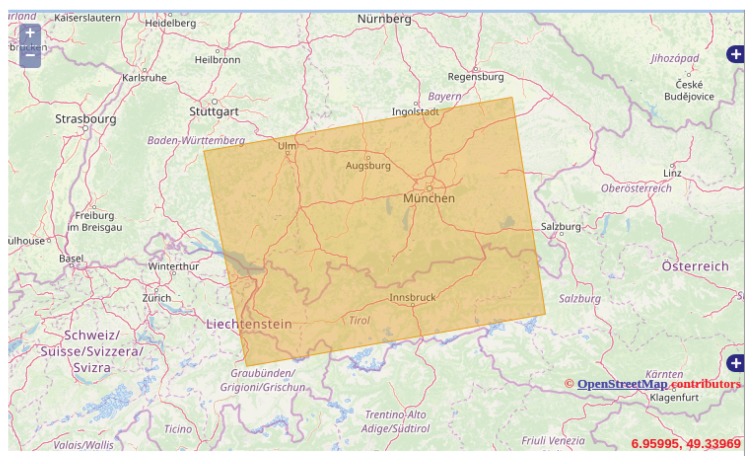
Sentinel 1 Interferometric Wide-Swath TOPS Mode imaged area: the yellow rectangle represents the investigated zone located in Southern Germany.

In particular, the investigated raw data include the Deutsche Zentrum für Luft-und Raumfahrt (DLR) calibration site, located in Southern Germany, where some corner reflectors are installed (see Figure 7).

This area has already been used for geometric, radiometric, and polarimetric calibration of several spaceborne SAR missions such as TerraSAR-X, TanDEM-X, S-1A and S-1B [50]. For the current study, two corner reflectors were used as reference targets.

**Figure 8:**
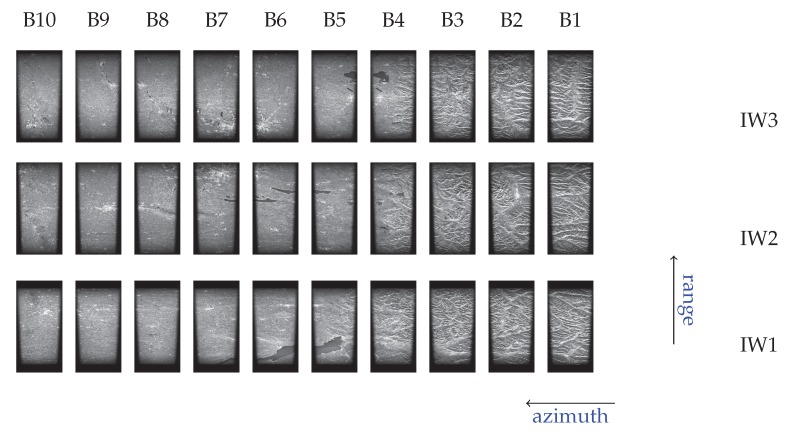
TOPS image focusing result: burst images sequence of the Sentinel-1B raw dataset acquired on 1 January 2019 on the area of interest.

Figure 8 shows the sequence of the focused TOPS burst images of Sentinel-1B raw dataset acquired on 1 January 2019 over the investigated area. The covered zone extended for about 250 Km × 200 Km. Please note that no antenna pattern correction has been applied. This image was used to analyze the response of the sensed corner reflectors to evaluate the developed focusing algorithm performance in term of spatial resolution and Peak Sidelobe Ratio (PSLR), as it will be described in the following.

In Table 3, the parameters relevant to the focused burst images are summarized. We highlight here that the output values of the implemented processor have been selected in order to be consistent with those of the image focused through the ESA processor generating the products available on the SciHub catalog [51]; to do this, an under-sampling factor $N_{under}=4$ has been used. On the other hand we remark that the azimuthal pixel spacing obtained through the developed focusing approach can be selected, depending on the user needs, by simply changing the $\tilde{r}$ value in Equation (35).

**Figure 9:**
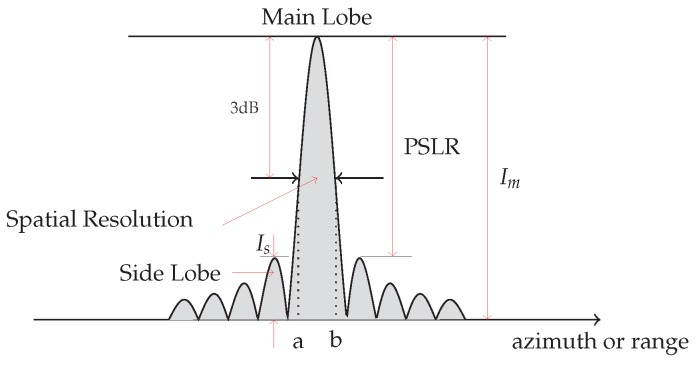
Impulse Response function and related parameters.

Figure 9 shows a one dimensional cut of the *Impulse Response Function* (IRF) of an ideal point target, along the range or azimuth direction and two quality parameters: the spatial resolution and the PSLR, respectively [2,3,4].

**Figure 10:**
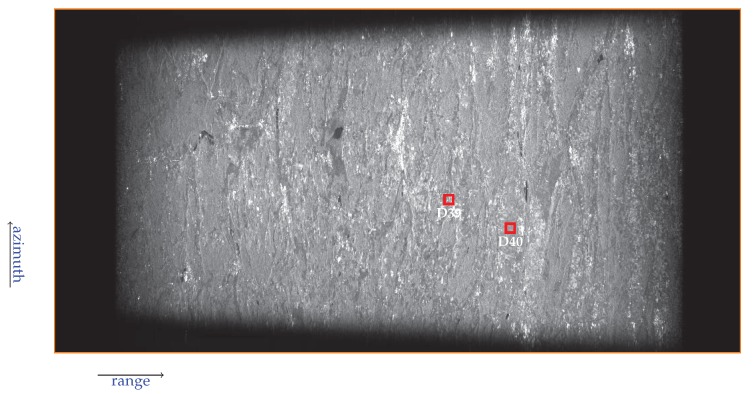
Focused image of burst 8 relevant to sub-swath 1: the position of two corner reflectors, referred to as D39 and D40 (see [50]), has been highlighted by the red squares.

Figure 10 shows the focused image relevant to burst 8 of the sub-swath 1, wherein the investigated corner reflectors have been highlighted.

**Figure 11:**
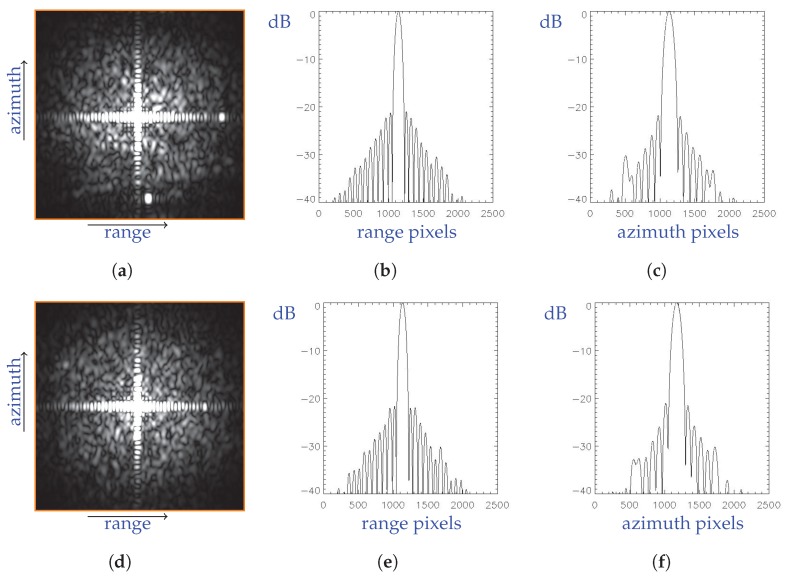
Corner reflectors within the burst 8 of sub-swath 1: (**a**) image, (**b**) IRF central cross section along range direction expressed in dB, (**c**) IRF central cross section along azimuth direction expressed in dB, for the corner reflector D39. (**d**) image, (**e**) IRF central cross section along range direction expressed in dB, (**f**) IRF central cross section along azimuth direction expressed in dB, for the corner reflector D40.

In Figure 11 the IRF central cross sections, along the range and azimuth direction, are displayed for the detected corner reflectors.

Quantitative results of the image quality analysis for the proposed focusing processing are listed in Table 4, where quality parameters for two corner reflectors within the focused burst 8 of sub-swath 1 have been considered. In particular, $\rho_{rg}$ and ${\hat{\rho }}_{rg}$ are the nominal and estimated range spatial resolutions, respectively, and $\rho_{az}$ and ${\hat{\rho }}_{az}$ are the corresponding values along the azimuth. Moreover, the nominal and estimated PLSR (PSLR and $\hat{PLSR}$, respectively) along range and azimuth has also presented.

Analogously, quantitative results of the image quality analysis on the corresponding product available through the ESA Schihub archive are listed in Table 5.

By comparing the achieved parameters for the presented focusing method (see Table 4 ) and the ESA products (see Table 5) with respect to the expected (nominal) values we find out a very good correspondence. Moreover, a slightly better azimuth resolution is achieved by the presented approach. Accordingly, we can promptly conclude that the proposed method has a very good performance. Please note also that to carry out the presented comparison an Hamming-0.75 weighting function has been applied to the burst image focused with the proposed method, in order to be consistent with the characteristics of the ESA images.

**Figure 12:**
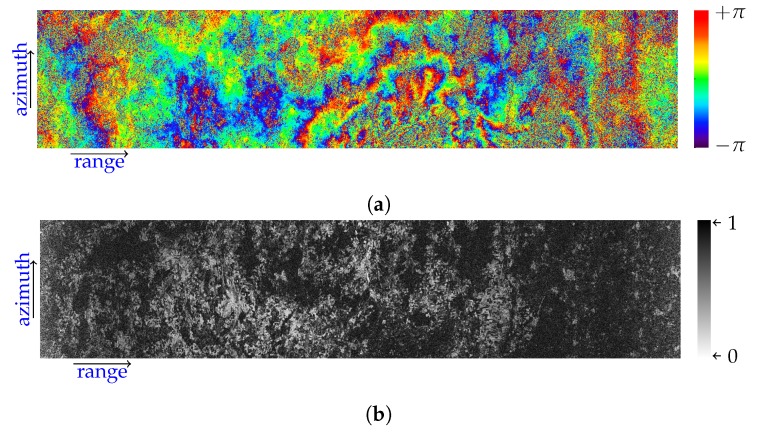
Burst interferogram (**a**) and the corresponding coherence (**b**) computed from the pair, focused through the presented approach, relevant to the S1B raw dataset acquired on August 23rd 2018 and the S1A raw dataset acquired six days later, over the DLR calibration site, shown in Figure 10

As final result, in order to verify the phase-preserving capability of the presented focusing method, we show in Figure 12 a burst interferogram and the corresponding coherence, obtained by processing, through the presented approach, an S1B raw dataset acquired on August 23rd 2018 and an S1A raw dataset acquired six days later; also in this case the considered burst is relevant to the DLR calibration site shown in Figure 10. The interferometric results presented in Figure 12, wherein the flat Earth phase component has been removed [41], fully confirm the phase-preserving capability of our approach.

## 5. Conclusions and Further Developments

In this work, an effective algorithm to focus SAR raw data acquired through the TOPS mode has been presented.

The proposed method allows the exploitation of conventional frequency domain and spectral analyses techniques, originally developed for processing Stripmap and ScanSAR raw data. In particular, although the presented technique is not dependent on the chosen frequency domain Stripmap processing approach, we consider the algorithm presented in [24,25,26,27]. In this case, the only modification with respect to the original technique is the inclusion, within the 2-D frequency domain focusing step, of a spurious azimuth chirp signal with a properly chosen constant rate. This allows us to avoid the extended azimuth zero padding, which would be needed to focus the raw data thought a straightforward Stripmap processing approach, and to exploit the high efficient SPECAN method [44] for the final azimuthal focusing of the TOPS raw data. Moreover, our approach also permits to easily achieve a constant and tunable output azimuth pixel size. This is a remarkable feature of the presented approach, with respect to the full-aperture TOPS-mode algorithms available in the existing literature.

Accordingly, the presented TOPS raw data focusing scheme is simple to be implemented and rather efficient, being based on FFTs and matrix multiplications, only. The experimental results, which have been carried out on a real Sentinel-1 SAR datasets relevant to the calibration site of DLR, confirmed the validity of the proposed focusing technique. In particular, the performance of the presented TOPS raw data focusing algorithm have been successfully tested by calculating some quality parameters, e.g., the spatial resolution and the PSLR of the SAR image impulsive response, in correspondence with available corner reflectors in the selected scene. Moreover, the phase-preserving capability of our approach has been demonstrated through the generation of interferometric products relevant to considered test site.

We further remark that the developed phase-preserving focusing algorithm can be effectively integrated in SAR processing toolboxes. Besides, the adopted focusing scheme is suitable for an easy implementation in a parallel computing environment, thus making profit from High-Performance Computing (HPC) architectures. Therefore, future actions will include the optimization of the different steps of the focusing processing based on their efficient implementation by using multi-core and GPUs parallelization [52,53,54]. To this end, the processing parts that can be parallelized both in terms of processes (multi-core parallelization for multiple bursts focusing processes) and in terms of data (parallelization within a single burst focusing process by using GPUs) will be identified. Accordingly, new modules (GPU kernels) making an efficient use of both cores and memory available on GPUs for parallel data processing, and modules that will exploit multithreading on the host machine (CPU), will be developed.

## Figures and Tables

**Table 1:** $N_{\mathrm{interp}}$ and $N_{\mathrm{focused}}$ values for IW1, IW2, and IW3 sub-swaths of the Sentinel-1 A/B sensors.

Sub-Swaths	$N_{\mathrm{interp}}$	$N_{\mathrm{focused}}$
IW1	7021	31919
IW2	7740	28575
IW3	7050	35040

**Table 2:** Sentinel-1A/B raw data parameters

Parameter	Swath	Value	Unit
Wavelength		0.055465756	m
Azimuth antenna size		12.300000	m
Number of sub-swaths		3	
Sampling Frequency	IW1	64,345,238	Hz
	IW2	54,595,960	Hz
	IW3	46,918,403	Hz
Range pixel spacing	IW1	2.3295620	m
	IW2	2.745555257	m
	IW3	3.194827944	m
Pulse Repetition Frequency	IW1	1717.1290	Hz
	IW2	1451.6271	Hz
	IW3	1685.8173	Hz
Azimuth pixel spacing	IW1	4.1779080	m
	IW2	4.9388437	m
	IW3	4.2459002	m
Angular Steering rate	IW1	1.5903688	degrees/s
	IW2	0.97986332	degrees/s
	IW3	1.3974408	degrees/s

**Table 3:** Sentinel-1A/B parameters of the focused burst images sequence.

Parameter	Swath	Value	Unit
Sampling Frequency	all	64345238	Hz
Range Pixel Spacing	all	2.3295620	m
Pulse Repetition Frequency	all	486.48631	Hz
Azimuth Pixel Spacing	all	14.713116	m

**Table 4:** Quality parameters for the corner reflectors focused through the presented algorithm within burst 8 of sub-swath 1: the nominal and estimated spatial resolutions along range ($\rho_{rg}$ and ${\hat{\rho }}_{rg}$, respectively), along azimuth ($\rho_{az}$ and ${\hat{\rho }}_{az}$, respectively), the nominal and estimated PLSR (PSLR and $\hat{PLSR}$, respectively) along range and azimuth are presented.

CRs	$\rho_{\mathrm{rg}}$	${\hat{\rho }}_{rg}$	$\rho_{\mathrm{az}}$	${\hat{\rho }}_{\mathrm{az}}$	PSLR	$\hat{\mathrm{PSLR}}$	$\hat{\mathrm{PSLR}}$
	(Nominal)		(Nominal)		(Nominal)	(Range)	(Azimuth)
	[m]	[m]	[m]	[m]	[dB]	[dB]	[dB]
D39	2.66	2.66	23.22	23.27	−21.21	−21.13	−21.92
D40		2.66		23.5		−21.87	−21.35

**Table 5:** Quality parameters for the corner reflectors focused through the ESA processor within burst 8 of sub-swath 1: the nominal and estimated spatial resolutions along range ($\rho_{rg}$ and ${\hat{\rho }}_{rg}$, respectively), along azimuth ($\rho_{az}$ and ${\hat{\rho }}_{az}$, respectively), the nominal and estimated PLSR (PSLR and $\hat{PLSR}$, respectively) along range and azimuth are presented.

CRs	$\rho_{\mathrm{rg}}$	${\hat{\rho }}_{rg}$	$\rho_{\mathrm{az}}$	${\hat{\rho }}_{\mathrm{az}}$	PSLR	$\hat{\mathrm{PSLR}}$	$\hat{\mathrm{PSLR}}$
	(Nominal)		(Nominal)		(Nominal)	(Range)	(Azimuth)
	[m]	[m]	[m]	[m]	[dB]	[dB]	[dB]
D39	2.66	2.66	23.22	24.88	−21.21	−21.16	−22.68
D40		2.66		24.65		−21.82	−21.85

## Abbreviations

The following abbreviations are used in this manuscript:

CSA	Chirp Scaling Algorithm
DC	Doppler Centroid
DLR	Deutsche Zentrum für Luft-und Raumfahrt
HPC	High-Performance Computing
IRF	Impulse Response Function
IWS	Interferometric Wide-Swath
PRF	Pulse Repetition Frequency
PSLR	Peak Side Lobe Ratio
RCM	Range Cell Migration
RDA	Range-Doppler Algorithm
SAR	Synthetic Aperture RADAR
TOPS	Terrain Observation by Progressive Scans
